# Bone microarchitecture in ankylosing spondylitis and the association with bone mineral density, fractures, and syndesmophytes

**DOI:** 10.1186/ar4368

**Published:** 2013-11-05

**Authors:** Eva Klingberg, Mattias Lorentzon, Jan Göthlin, Dan Mellström, Mats Geijer, Claes Ohlsson, Elizabeth J Atkinson, Sundeep Khosla, Hans Carlsten, Helena Forsblad-d’Elia

**Affiliations:** 1 Department of Rheumatology and Inflammation Research, Sahlgrenska Academy at the University of Gothenburg, Gothenburg, Sweden; 2 Centre for Bone and Arthritis Research, Institute of Medicine, Sahlgrenska Academy at the University of Gothenburg, Gothenburg, Sweden; 3 Department of Radiology, Sahlgrenska Hospital/Mölndal, Sahlgrenska Academy at the University Hospital/Mölndal, Gothenburg, Sweden; 4 Centre for Medical Imaging and Physiology, Skåne University Hospital, Lund, Lund University, Lund, Sweden; 5 College of Medicine, Mayo Clinic, Rochester, MN, USA; 6 College of Physiology and Medicine, Mayo Clinic, Rochester, MN, USA

## Abstract

**Introduction:**

Osteoporosis of the axial skeleton is a known complication of ankylosing spondylitis (AS), but bone loss affecting the peripheral skeleton is less studied. This study on volumetric bone mineral density (vBMD) and bone microarchitecture in AS was conducted to compare peripheral vBMD in AS patients with that in healthy controls, to study vBMD in axial compared with peripheral bone, and to explore the relation between vertebral fractures, spinal osteoproliferation, and peripheral bone microarchitecture and density.

**Methods:**

High-resolution peripheral quantitative computed tomography (HRpQCT) of ultradistal radius and tibia and QCT and dual-energy x-ray absorptiometry (DXA) of lumbar spine were performed in 69 male AS patients (NY criteria). Spinal radiographs were assessed for vertebral fractures and syndesmophyte formation (mSASSS). The HRpQCT measurements were compared with the measurements of healthy controls.

**Results:**

The AS patients had lower cortical vBMD in radius (*P* = 0.004) and lower trabecular vBMD in tibia (*P* = 0.033), than did the controls. Strong correlations were found between trabecular vBMD in lumbar spine, radius (r_S_ = 0.762; *P* < 0.001), and tibia (r_S_ = 0.712; *P* < 0.001).

When compared with age-matched AS controls, patients with vertebral fractures had lower lumbar cortical vBMD (-22%; *P* = 0.019), lower cortical cross-sectional area in radius (-28.3%; *P* = 0.001) and tibia (-24.0%; *P* = 0.013), and thinner cortical bone in radius (-28.3%; *P* = 0.001) and tibia (-26.9%; *P* = 0.016).

mSASSS correlated negatively with trabecular vBMD in lumbar spine (r_S_ = -0.620; *P* < 0.001), radius (r_S_ = -0.400; p = 0.001) and tibia (r_S_ = -0.475; p < 0.001) and also with trabecular thickness in radius (r_S_ = -0.528; *P* < 0.001) and tibia (r_S_ = -0.488; *P* < 0.001).

Adjusted for age, syndesmophytes were significantly associated with decreasing trabecular vBMD, but increasing cortical vBMD in lumbar spine, but not with increasing cortical thickness or density in peripheral bone. Estimated lumbar vBMD by DXA correlated with trabecular vBMD measured by QCT (r_S_ = 0.636; *P* < 0.001).

**Conclusions:**

Lumbar osteoporosis, syndesmophytes, and vertebral fractures were associated with both lower vBMD and deteriorated microarchitecture in peripheral bone. The results indicate that trabecular bone loss is general, whereas osteoproliferation is local in AS.

## Introduction

Ankylosing spondylitis (AS) is a chronic inflammatory rheumatic disease predominantly affecting the sacroiliac joints and the vertebral column. The disease often leads to the formation of spinal syndesmophytes and impaired back mobility, especially in male patients. Patients with AS have, in comparison with the general population, an increased risk of developing osteoporosis and fractures, especially vertebral fractures, but also other fractures, including hip fractures [1-6]. Male sex, old age, long disease duration, elevated inflammatory parameters, advanced chronic AS-related changes in the spine, and poor back mobility have been identified as risk factors for osteoporosis and vertebral fractures [7-13]. The mechanisms behind inflammation, new-bone formation and osteoporosis in AS are incompletely understood.

Measurements of lumbar area bone mineral density (aBMD) with dual-energy x-ray absorptiometry (DXA) in the anteroposterior (AP) projection are unreliable in AS, because of the spinal osteoproliferation [13-15]. Furthermore, prospective studies of the general population have shown that aBMD identifies only 20% of men who will later sustain a fracture [16,17]. Bone strength depends partly on bone mineral content, but also on the bone geometry and microarchitecture of cortical and trabecular bone [18-20]. Quantitative computed tomography (QCT) has the advantage of assessing volumetric BMD (vBMD) in the lumbar spine in cortical and trabecular bone separately, without including areas of hyperostosis in the measurements [15].

The development of high-resolution peripheral quantitative computed tomography (HRpQCT) has enabled us to study bone in great detail, without the need of biopsies. Although several studies exist on osteoporosis in AS, the knowledge of the bone microarchitecture in the disease is still sparse. The evolving technique of HRpQCT offers new possibilities to study further the intricate association between new bone formation and osteoporosis in AS and also to follow up the effects of treatment for osteoporosis and inflammation on bone tissue. This is, to our best knowledge, the first study on bone microarchitecture in AS with HRpQCT.

The aims of the present study were to (a) study peripheral vBMD in AS patients in comparison with healthy controls, (b) compare vBMD of trabecular and cortical bone in the axial and peripheral skeleton, (c) explore the relation between bone microarchitecture and presence of vertebral fractures and syndesmophytes, and (d) compare lumbar BMD measured with QCT and DXA in the AP and lateral projections.

## Methods

### Patients

In total, 69 male AS patients who had been included in a larger study on osteoporosis in the west of Sweden were randomized in an age-adjusted algorithm also to take part in the current study on HRpQCT and QCT. All AS patients registered at the Rheumatology Clinic at Sahlgrenska University Hospital in Gothenburg, and the Rheumatology Clinics at Borås and Alingsås county hospitals had been invited to take part in the initial study. The inclusion of the 204 patients in the initial study has been described in detail [13]. In summary, all included patients met the modified New York criteria for AS [21] .Exclusion criteria were psoriasis, inflammatory bowel disease, dementia, and difficulties in understanding Swedish. Written informed consent was obtained from all patients. The study was approved by the Regional Ethical Review Board in Gothenburg and carried out in accordance with the Helsinki declaration.

### Healthy controls

The HRpQCT results regarding trabecular and cortical vBMD of the AS patients were compared with the results of a control group consisting of 68 healthy individuals measured with the same type of XtremeCT at the Mayo Clinic in Rochester, Olmsted County, Minnesota, USA. The healthy controls were matched for age, height, weight, and race. No control could be found for the youngest patient, who thus was excluded from these calculations.

### High-resolution peripheral quantitative computed tomography

Bone microarchitecture was examined by using an HRpQCT device (XtremeCT; Scanco Medical AG, Brüttisellen, Switzerland) in the nondominant ultradistal radius and tibia. The patient’s forearm and leg were immobilized in especially designed carbon-fiber shells (Scanco Medical) to prevent movement during the procedure.

The quality of the measurements was assessed by using a 5-point scale recommended by the manufacturer (1, excellent; 2, good; 3, acceptable; 4, unacceptable; 5, poor). Only examinations with quality grades 1 through 3 were included in the study, whereas grades 4and 5 were excluded, mostly because of motion artefacts. Totally 12 examinations of the radius had to be repeated because of to movement artifacts. Seven patients had measurements of the ultradistal radius with unacceptable quality and were thus excluded, whereas all tibia measurements were included.

The volumes of interest (VOIs), 9-mm sections of radius and tibia, were examined in 110 parallel slices (voxel size, 82 μm), generating a 3D representation of the bone. The first CT slices started 9.5 mm and 22.5 mm proximal to a reference line manually placed at the center of the end plate of the distal radius and tibia, respectively, and continued proximally.

The VOIs were automatically separated into a trabecular and a cortical region. With previously described data-extracting procedures, the following parameters for the trabecular and cortical bone were obtained: trabecular volumetric BMD (DTrab; mg/cm^3^), trabecular bone volume/total volume (BV/TV; %), trabecular number (TbN; per mm), trabecular thickness (TbTh; μm), trabecular separation or spacing (TbSp; μm), cortical volumetric BMD (DCort; mg/cm^3^), cortical bone cross-sectional area (CortCSA; mm^2^), cortical periosteal circumference (CortPm; mm), and cortical thickness (CortTh; μm) [22-26]. DTrab, DCort, TbN, CortCSA, and CortPm were measured directly, and the other parameters were derived.

The coefficients of variation (CVs) for repeated measurements by using the XtremeCT apparatus in Gothenburg ranged between 0.3% and 3.9% of the radius and from 0.1% to 1.6% of the tibia. The same device, software, and operator were used throughout the study.

The software Autocontouring and Eval Crtx 6× software, provided by Scanco Incorporated in the manufacturer’s Image Processing Language (IPL) software (μCT Evaluation Program v6; Scanco Medical AG, Brüttisellen, Switzerland) was used to assess cortical bone microstructure of the ultradistal radius and tibia. The cortical compartment in the VOI was detected automatically by identifying the endosteal and periosteal contours. All void voxels within the cortical compartment were identified, and the images were digitally superimposed, generating a refined cortical compartment region in the VOI. The Haversian canals were distinguished from artefacts because of surface roughness, transcortical foramens, or erosions. With this method, cortical porosity (CtPo; %) and mean cortical pore diameter (CtPoDiam; μm) were obtained [22,23,25]. The CVs for porosity were 15.9% at the radius and 5.5% at the tibia, and the CVs for mean cortical pore diameter were 6.0% at the radius and 3.9% at the tibia.

The healthy control group was assessed with the same type of XtremeCT at the Mayo Clinic in Rochester. A scan phantom for the vBMD measurements was sent from Gothenburg to Rochester for cross-calibration of the XtremeCT devices. Linear regression between the phantom measurements in Gothenburg and Rochester generated a formula

$$\left(\left(X\times 0.995\right)+1.148\right)$$

by which the values from Rochester were subsequently adjusted. The phantom was not designed for cross-calibration of microarchitectural parameters; hence those parameters could not be compared in the patients and controls.

### Quantitative computed tomography

Lumbar volumetric BMD was measured in the vertebrae L1 through L4 by using a QCT scanner (Siemens Somatom Sensation 16 with application Syngo Osteo CT; Siemens AG, Munich, Germany). Volumetric BMD (vBMD; mg/cm^3^) was assessed separately in the cortical and the trabecular bone in 10-mm-thick slices of each vertebra. All patients were scanned together with a water- and bone-equivalent calibration phantom placed below the patients along with an interpositioned gel pad to prevent artefacts and air gaps. The BMD results of the patients were compared with the reference population database of the CT scanner software, including 135 male and 139 female European subjects, 20 through 80 years of age.

### Dual-energy x-ray absorptiometry

BMD was measured with DXA (Hologic Discovery A; Hologic Inc., Bedford, MA, USA) in the nondominant forearm (total radius, radius 1/3) and hip (total hip, femoral neck) and in the lumbar spine (in AP L1 through L4 and lateral L2 through L4 projection) with estimation of lumbar vBMD.

### Radiography

Lateral radiographs of the spinal column were taken to study the presence of vertebral fractures in the thoracic and lumbar spine by using the Genant score, which scores vertebrae on visual inspection as normal, mildly, moderately, or severely deformed (grades 0 to 3) [27]. All vertebral fractures (Genant score, 1 through 3) were included in the calculations. The presence of chronic AS changes in the cervical and lumbar spine was assessed by using the modified Stoke Ankylosing Spondylitis Spine Score (mSASSS) [28] .The score grades the anterior vertebral corners with 0 through 3 points each (0, normal; 1, erosion, sclerosis, or squaring; 2, syndesmophyte; or 3, bridging syndesmophyte). The scoring scale ranges from 0 to 72.

### Statistical analysis

Statistical analyses were performed by using PASW Statistics 18.0 (SPSS Inc., IBM, Chicago, IL, USA). Descriptive statistics are presented as median and range and/or mean and standard deviation (SD). The Mann–Whitney *U* test, *t* test, or the χ^2^ test was used to compare variables as appropriate. Correlations were calculated by using the Spearman correlation (r_s_). Logistic regressions with a forward conditional method were run with the presence of a syndesmophyte (yes/no) and vertebral fracture (yes/no) as the binary outcome. All tests were two-tailed, and *P* < 0.05 was considered statistically significant.

## Results

### Patients

In total, 69 male AS patients were included in the study. The characteristics of the patients are presented in Table 1.

**Table 1:** Characteristics of 69 male patients with ankylosing spondylitis in western Sweden

	***n *** **(%)**	**Median (range)**	**Mean ± SD**
Age, years		48 (17, 78)	49 ± 15
Years since symptom onset		20 (2, 55)	23 ± 14
Present or past iritis	37 (54)		
Present or past synovitis	37 (54)		
Patients with a vertebral fracture	8 (12)		
mSASSS		8 (0, 72)	19 ± 21
BASMI, score		3.0 (1.0, 7.2)	3.2 ± 1.6
BASDAI, score		2.7 (0.2, 7.9)	3.1 ± 2.0
ASDAS, score		2.1 (1.0, 4.4)	2.3 ± 0.9
BAS-G		2.2 (0, 9.8)	3.0 ± 2.7
BASFI, score		2.0 (0, 8.7)	2.6 ± 2.2
ESR, mm/h		10 (2, 73)	14 ± 12
CRP, mg/L		5 (3, 71)	9 ± 12
HLA-B27 positive	65 (94)		
Patients taking NSAID	52 (75)		
Patients taking glucocorticoid	1 (1)		
Patients taking DMARD	21 (30)		
Methotrexate	13 (19)		
Sulfasalazine	5 (7)		
TNF inhibitors	15 (22)		

BASDAI, Bath Ankylosing Spondylitis Disease Activity Index; BASFI, BAS Functional Index; BASG1, BAS Patient Global score; BASMI, BAS Metrology Index; ESR, erythrocyte sedimentation rate; CRP, C-reactive protein; NSAID, nonsteroidal antiinflammatory drug; DMARD, disease-modifying antirheumatic drug; TNF, tumor necrosis factor; mSASSS, modified Stoke Ankylosing Spondylitis Spine Score.

### vBMD measured with HRpQCT in the AS patients compared with the healthy controls

The AS patients had, in comparison with the controls, significantly lower vBMD in cortical bone of the ultradistal radius (*P* = 0.007) and in trabecular bone of the ultradistal tibia (*P* = 0.033) (Table 2). The AS patients also had lower weight and body mass index (BMI) than the controls, but the differences did not reach a level of significance. Weight and BMI were, however, not correlated with trabecular or cortical vBMD in the ultradistal radius or tibia. (Correlation coefficients ranged from -0.082 to 0.100, and *P* values ranged from 0.245 to 0.853).

**Table 2:** Comparison of the demographics and HRpQCT parameters in the AS patients and healthy age-matched controls

		**AS patients**	**Healthy controls**	**Significance**
				***P *** **value**
Demographics	Male %	100	100	
	Age (years)	49 ± 14	49 ± 14	0.993
	BMI kg/m^2^	26.6 ± 4.4	27.8 ± 4.6	0.117
	Height cm	177 ± 7	179 ± 7	0.358
	Weight kg	84 ± 14	89 ± 16	0.052
HRpQCT radius	DTrab mg/cm^3^	181 ± 39	187 ± 39	0.460
	DCort mg/cm^3^	850 ± 55	874 ± 42	0.007
HRpQCT tibia	DTrab mg/cm^3^	187 ± 35	201 ± 41	0.033
	DCort mg/cm^3^	844 ± 53	845 ± 44	0.944

The youngest AS patient was excluded from this analysis because of lack of a healthy control. Calculations on the tibia were made on 68 patients and controls and on radius of 61 patients and controls. The HRpQCT measurements of the radius from seven AS patients had to be excluded towing to motion artefacts. BMI, body mass index; DCort, vBMD of cortical peripheral bone; DTrab, vBMD of trabecular peripheral bone; HRpQCT, high-resolution peripheral quantitative computed tomography.

### Correlation between spinal vBMD measured with QCT and peripheral bone microarchitecture measured with HRpQCT

**Figure 1:**
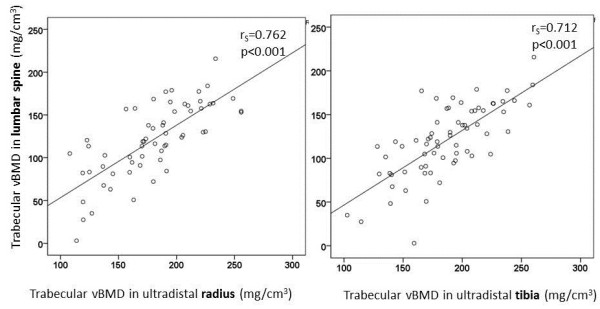
The correlations between trabecular volumetric BMD in the lumbar spine, ultradistal radius, and tibia.

**Figure 2:**
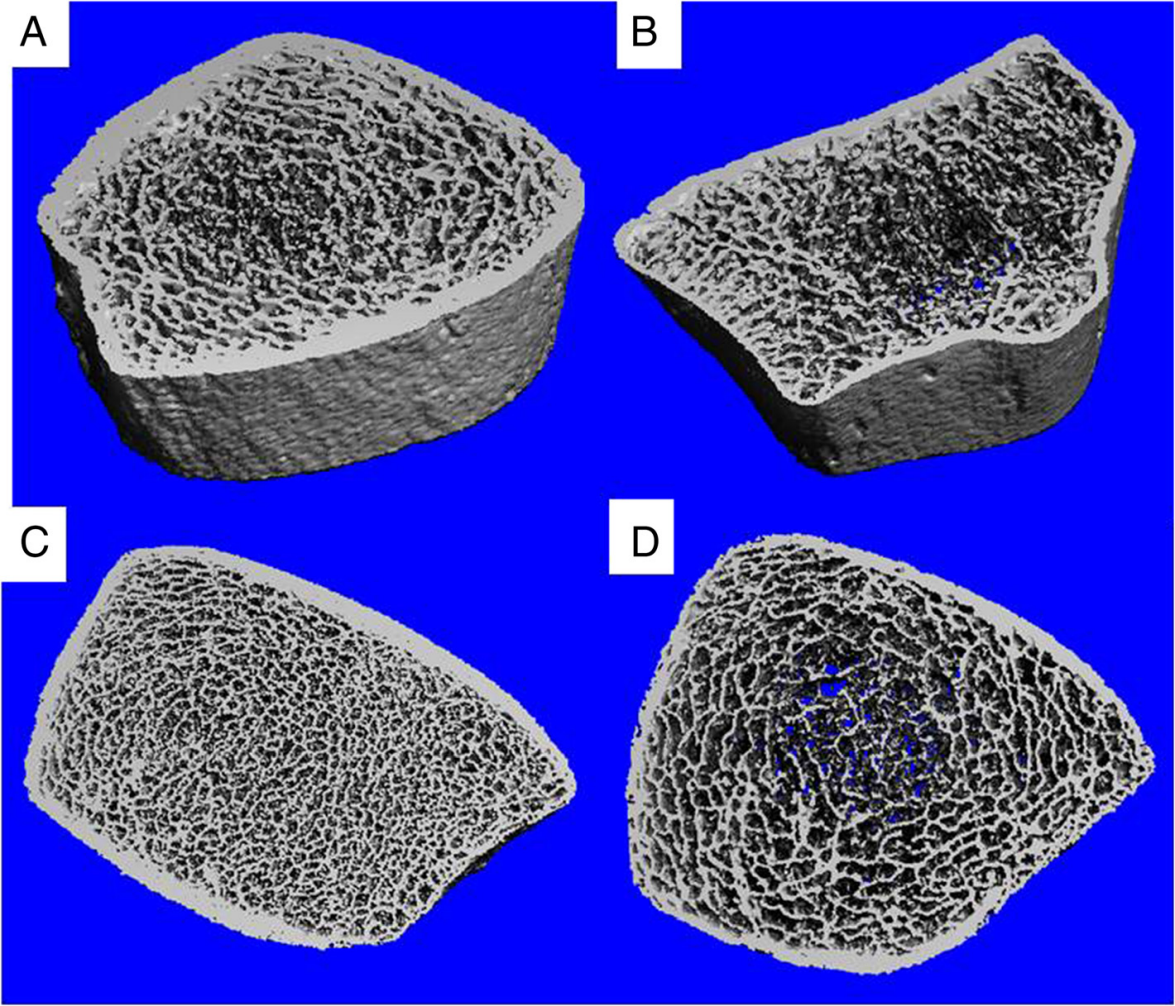
**HRpQCT images of ultradistal radius and tibia. (A)** 3D representation of ultradistal tibia. AS patient with normal BMD. **(B)** 3D representation of ultradistal radius. AS patient with osteoporosis. **(C)** 2D representation of ultradistal tibia. AS patient with normal BMD. **(D)** 2D representation of ultradistal tibia. AS patients with lumbar osteoporosis. Note the low trabecular number and thickness, high trabecular separation, and low cortical thickness.

Strong correlations were found between trabecular vBMD in the lumbar spine, ultradistal radius (r_S_ = 0.762; *P* < 0.001) and tibia (r_S_ = 0.712; *P* < 0.001), but the cortical vBMD of the spine and peripheral skeleton were not significantly correlated (Figure 1 and Table 3). Low-lumbar trabecular vBMD was also significantly correlated with parameters indicating poor bone microarchitecture, such as thinner trabeculae, lower trabecular number, thinner cortex, lower cortical vBMD, and increased cortical porosity (Figure 2 and Table 3). The results thus indicated a link between trabecular bone loss in the axial and peripheral skeleton in AS.

**Table 3:** The correlation (Spearman rho) between the HRpQCT parameters, age, lumbar QCT, and mSASSS in the AS patients

**HRpQCT radius**	**DTrab (mg/cm**^**3**^**)**	**BV/TV (%)**	**Tbth (μm)**	**TbN (per mm)**	**TbSp (μm)**	**DCort (mg/cm**^**3**^**)**	**CortTh (μm)**	**CtPo (%)**	**CtPoDiam (μm)**
**Age**	-0.471	-0.473	-0.507	-0.040	0.166	-0.337	-0.228	0.540	0.055
	***P*** **< 0.001**	***P*** **< 0.001**	***P*** **< 0.001**	*P* = 0.755	*P* = 0.198	***P*** **= 0.007**	*P* = 0.075	***P*** **< 0.001**	*P* = 0.663
**Lumb QCT**	0.762	0.763	0.737	0.106	-0.304	0.425	0.420	-0.480	- 0.136
**Trab vBMD**	***P*** **< 0.001**	***P*** **< 0.001**	***P*** **< 0.001**	*P* = 421	***P*** **= 0.018**	***P*** **= 0.001**	***P*** **= 0.001**	***P*** **< 0.001**	*P* = 0.290
**Lumb QCT**	0.296	0.298	0.152	0.296	- 0.341	0.123	0.152	0.032	0.006
**Cort vBMD**	***P*** **= 0.021**	***P*** **= 0.021**	*P* = 0.246	***P*** **= 0.022**	***P*** **= 0.008**	*P* = 0.348	*P* = 247	*P* = 0.805	*P* = 0.961
**Msasss**	- 0.400	- 0.400	- 0.528	0.077	0.039	- 0.192	- 0.196	0.352	0.022
	***P*** **= 0.001**	***P*** **= 0.001**	***P*** **< 0.001**	*P* = 0.552	*P* = 0.761	*P* = 0.135	*P* = 0.128	***P*** **= 0.004**	*P* = 0.861
**HRpQCT tibia**	**DTrab**	**BV/TV**	**Tbth**	**TbN**	**TbSp**	**DCort**	**CortTh**	**CtPo**	**CtPoDiam**
	(mg/cm^3^)	(%)	(μm)	(mm^-1^)	(μm)	(mg/cm^3^)	(μm)	(%)	(μm)
**Age**	- 0.470	- 0.471	- 0.479	- 0.185	0.246	- 0.436	- 0.202	0.521	0.210
	***P*** **< 0.001**	***P*** **< 0.001**	***P*** **< 0.001**	*P* = 0.127	***P*** **= 0.042**	***P*** **< 0.001**	*P* = 0.096	***P*** **< 0.001**	0.084
**Lumb QCT**	0.712	0.714	0.523	0.363	- 0.443	0.454	0.403	- 0.496	- 0.437
**Trab vBMD**	***P*** **< 0.001**	***P*** **< 0.001**	***P*** **< 0.001**	***P*** **= 0.003**	***P*** **< 0.001**	***P*** **< 0.001**	***P*** **= 0.001**	***P*** **< 0.001**	***P*** **< 0.001**
**Lumb QCT**	0.336	0.336	0.027	0.356	- 0.367	0.135	0.151	- 0.102	- 0.162
**Cort vBMD**	***P*** **= 0.005**	***P*** **= 0.005**	*P* = 0.827	***P*** **= 0.003**	***P*** **= 0.002**	*P* = 0.276	*P* = 0.223	*P* = 0.410	*P* = 0.191
**mSASSS**	- 0.475	- 0.475	- 0.488	- 0.105	0.176	- 0.315	-0.161	0.363	0.169
	***P*** **< 0.001**	***P*** **< 0.001**	***P*** **< 0.001**	*P* = 0.392	*P* = 0.148	***P*** **= 0.008**	*P* = 0.186	***P*** **= 0.002**	*P* = 0.169

BV/TV, trabecular bone volume fraction; Cort, cortical; DCort, vBMD of cortical peripheral bone; DTrab, vBMD of trabecular peripheral bone; CortTh, cortical thickness peripheral bone; CtPo, cortical porosity; CtPoDiam, mean cortical pore diameter; HRpQCT, high-resolution peripheral quantitative computed tomography; Lumb, lumbar; TbN, trabecular number peripheral bone; TbSp, trabecular separation peripheral bone; Tbth,trabecular thickness peripheral bone; Trab, trabecular; vBMD, volumetric bone mineral density; QCT, quantitative computed tomography.

Trabecular and cortical vBMD were significantly correlated in the ultradistal radius (r_S_ = 0.466; *P* < 0.001) and tibia (r_S_ = 0.308; *P* < 0.010), but the correlation did not reach the level of significance (r_S_ = 0.230; *P* = 0.061) in the lumbar spine.

### Vertebral fractures in relation to bone mineral density and peripheral bone microarchitecture

Vertebral fractures were diagnosed in eight (12%) patients, the youngest patient being 31, and the oldest, 71 years old. Twelve vertebral fractures with Genant score grade 1 and two with grade 2 were diagnosed in the study group. Age, disease duration, and mSASSS score were not significantly different between patients with or without a vertebral fracture.

Each patient with a vertebral fracture was compared with two age-matched AS controls from the same cohort (Table 4). The patients with a vertebral fracture had significantly lower cortical lumbar vBMD measured with QCT compared with the age-matched nonfractured patients. The following DXA parameters were also significantly lower in the patients with a vertebral fracture: AP and lateral lumbar BMD, lumbar vBMD, and BMD of the hip (femoral neck and total hip).

**Table 4:** BMD and microarchitectural parameters in AS patients with a vertebral fracture compared with age-matched AS patients without a vertebral fracture

		**Patients without a vertebral fracture (mean ± SD)**	**Patients with a vertebral fracture (mean ± SD)**	**Difference%**	***P *** **value**
		***n*** **= 16**	***n*** **= 8**		
	Age (years)	55.9 ± 12.4	55.6 ± 12.8	-0.01	0.955
QCT lumbar spine	Trab vBMD (mg/cm^3^)	117.17 ± 34.35	92.09 ± 40.94	-21.4	0.128
	Cort vBMD (mg/cm^3^)	366.11 ± 81.62	285.45 ± 52.77	-22.0	0.019
DXA BMD	AP lumbar (g/cm^2^)	1.10 ± 0.11	0.98 ± 0.13	-10.9	0.018
	Lat lumbar (g/cm^2^)	0.80 ± 0.11	0.68 ± 0.10	-15.0	0.021
	Lumb vBMD (g/cm^3^)	0.20 ± 0.03	0.17 ± 0.03	-15.0	0.048
	Femoral neck (g/cm^2^)	0.80 ± 0.11	0.66 ± 0.12	-17.5	0.009
	Total hip (g/cm^2^)	1.00 ± 0.10	0.83 ± 0.13	-17.0	0.002
	Radius 1/3 (g/cm^2^)	0.79 ± 0.05	0.78 ± 0.08	-1.3	0.672
	Total radius (g/cm^2^)	0.66 ± 0.05	0.62 ± 0.07	-6.1	0.188
HRpQCT	DTrab (mg/cm^3^)	195.42 ± 40.31	147.20 ± 27.10	-24.7	0.010
Radius					
	BV/TV (%)	16.3 ± 3.4	12.3 ± 2.3	-25.0	0.010
	Tbth (μm)	83.1 ± 19.9	65.1 ± 7.9	-12.5	0.034
	TbN (per mm)	1.98 ± 0.26	1.88 ± 0.23	-5.1	0.388
	TbSp (μm)	430.0 ± 66.1	473.1 ± 64.7	+9.3	0.169
	DCort (mg/cm^3^)	862.81 ± 38.45	810.70 ± 68.01	-6.0	0.032
	CortTh (μm)	901 ± 163	637 ± 181	-28.9	0.003
	CortCSA (mm^2^)	76.81 ± 11.63	55.11 ± 14.17	-28.3	0.001
	CtPo (%)	2.65 ± 0.97	2.39 ± 1.07	-9.8	0.548
	CtPoDiam (μm)	172 ± 18	165 ± 15	-4.1	0.548
HRpQCT	DTrab (mg/cm^3^)	189.52 ± 25.76	155.59 ± 39.46	-17.9	0.052
Tibia					
	BV/TV (%)	15.8 ± 2.1	13.0 ± 3.3	-18.8	0.050
	Tbth (μm)	78.6 ± 9.4	68.4 ± 9.4	-12.5	0.020
	TbN (per mm)	2.01 ± 0.18	1.88 ± 0.26	-6.4	0.149
	TbSp (μm)	421.9 ± 44.8	474.6 ± 89.1	+14.3	0.150
	DCort (mg/cm^3^)	848.24 ± 51.18	810.19 ± 83.08	-4.5	0.263
	CortTh (μm)	1,303 ± 297	946 ± 352	-26.9	0.016
	CortCSA (mm^2^)	151.94 ± 26.71	115.50 ± 38.91	-24.0	0.013
	Ct Po (%)	7.27 ± 2.94	7.54 ± 3.27	+3.7	0.881
	CtPoDiam (μm)	189 ± 16	194 ± 17	+2.6	0.417

Difference (%), mean of fractured – mean of nonfractured/mean of nonfractured; BMD, bone mineral density; BV/TV, trabecular bone volume fraction; Cort, cortical; DCort, vBMD of cortical peripheral bone; DTrab, vBMD of trabecular peripheral bone; DXA, dual-energy x-ray absorptiometry; CortCSA, cortical cross-sectional area peripheral bone; CortTh,cortical thickness peripheral bone, CtPo = cortical porosity; CtPoDiam, mean cortical pore diameter; HRpQCT, high-resolution peripheral quantitative computed tomography; TbN, trabecular number peripheral bone; TbSp, trabecular separation peripheral bone; Tbth, trabecular thickness peripheral bone; Trab, trabecular; vBMD, volumetric BMD; QCT, quantitative computed tomography.

No significant difference in BMD was found in the forearm measured by DXA between the groups. When measured with HRpQCT, the patients with a vertebral fracture, however, displayed significantly lower trabecular and cortical vBMD in the ultradistal radius and, in addition, lower trabecular thickness, cortical thickness, and cross-sectional area in both the ultradistal radius and tibia, thus indicating deteriorated peripheral bone microarchitecture in the fractured patients in comparison with the age-matched AS controls. The greatest differences between the fractured and nonfractured patients were found in cortical thickness and cortical cross-sectional area (Table 4).

Multiple logistic regression was run with vertebral fracture as binary outcome. In the first model, in which age, mSASSS, and trabecular and cortical lumbar vBMD were entered as covariates, decreasing cortical lumbar vBMD (B = -0.023; *P* = 0.015; OR, 0.977; 95% CI, 0.959 to 0.996), and increasing mSASSS (B = 0.042; *P* = 0.049; OR, 1.04; 95% CI, 1.0003 to 1.087) were independently associated with the presence of a vertebral fracture.

In the second model, in which the HRpQCT parameters were directly measured and not derived (DTrab, DCort, TbN, CortCSA, and CortPm) were additionally entered, only decreasing cortical cross-sectional area of the tibia (B = -0.063; *P* = 0.008; OR, 0.939; 95% CI, 0.897 to 0.984) remained significantly associated with the presence of a vertebral fracture.

### Syndesmophyte formation in relation to spinal vBMD measured with QCT and peripheral bone microarchitecture

Increasing mSASSS correlated significantly with increasing age (r_S_ = 0.546; *P* < 0.001) and decreasing trabecular vBMD in the lumbar spine (r_S_ = -0.620; *P* < 0.001). In addition, mSASSS correlated with increasing cortical porosity and decreasing trabecular thickness and vBMD of trabecular and cortical bone in the periphery (Table 3).

In 39 patients, radiographs of the cervical and lumbar spines revealed at least one syndesmophyte or bridging syndesmophyte, whereas nine patients had a disease restricted to the sacroiliac joints (mSASSS = 0), and 21 patients had only the presence of erosions, sclerosis, or squaring at the vertebral corners.

The patients with at least one syndesmophyte had, in comparison with patients without syndesmophytes, significantly older age (54 ± 12 versus 42 ± 15 years; *P* = 0.001), thinner trabeculae in the ultradistal radius (0.071 ± 0.016 versus 0.085 ± 0.014, *P* < 0.001) and tibia (0.072 ± 0.013 versus 0.081 ± 0.008; *P* = 0.001) and lower trabecular vBMD in the lumbar spine (100 ± 36 versus146 ± 33; *P* < 0.001), ultradistal radius (171 ± 42 versus 196 ± 29; *P* = 0.013) and tibia (175 ± 37 versus 205 ± 27; *P* < 0.001).

In a multiple logistic regression model with the presence of at least one syndesmophyte as the binary outcome and adjusting for age, decreasing lumbar trabecular vBMD (B = -0.058; *P* < 0.001; OR = 0.943; 95% CI, 0.917 to 0.970), but increasing lumbar cortical vBMD (B = 0.019; *P* = 0.016; OR, 1.019; 95% CI, 1.004 to 1.035) remained independently associated with syndesmophyte formation. None of the HRpQCT parameters was significantly associated with presence of syndesmophytes after adjusting for age in logistic regression. Covariates in the regression model were age, trabecular and cortical lumbar vBMD, and the HRpQCT parameters, which were directly measured and not derived (DTrab, DCort, TbN, CortCSA, and CortPm).

### Comparisons of BMD measured with QCT and with DXA in AP and lateral projections in the lumbar spine

QCT revealed significantly more cases with T score ≤2.5 SD (*n* = 26; 38%) and T score ≤1.0 SD (*n* = 21; 30%) than AP DXA, showing considerably less osteoporosis (*n* = 4; 6%) and osteopenia (*n* = 13; 19%; *P* < 0.001). No reference database values were available for lateral lumbar DXA and DXA vBMD lumbar spine.

The patients with at least one syndesmophyte had significantly reduced Z scores of lumbar vBMD measured with QCT (mean Z score, -0.624 ± 1.186; *P* = 0.003), whereas AP lumbar DXA showed no significant reduction of the Z scores of lumbar BMD in patients with or without syndesmophytes, thus indicating that the QCT results were less affected by syndesmophyte formation than was the AP lumbar DXA.

Correlation analyses between lumbar QCT and lumbar DXA demonstrated that QCT trabecular vBMD had the strongest correlation with DXA vBMD (r_S_ = 0.636; *P* < 0.001) followed by lateral BMD (r_S_ = 0.537; *P* < 0.001) and AP BMD (r_S_ = 0.380; *P* = 0.002). QCT cortical vBMD correlated with DXA in the following way: lateral BMD (r_S_ = 0.595; *P* < 0.001), AP BMD (r_S_ = 0.541; *P* = 0.002) and vBMD (r_S_ = 0.431; *P* < 0.001).

## Discussion

In the present study, we used new imaging techniques, peripheral HRpQCT and lumbar QCT, to investigate the relation between osteoporosis, osteoproliferation, and fractures in the spine and morphology and bone density in the peripheral skeleton.

When measured with HRpQCT, the patients with AS had lower vBMD in the peripheral skeleton than did healthy controls; the AS patients had significantly lower vBMD in the cortical bone of the radius and the trabecular bone of the tibia. The controls were slightly heavier than the AS patients, but it seems unlikely that this would explain the difference, because vBMD in ultradistal radius and tibia were not correlated with weight or BMI.

The HRpQCT measurements of the radius from seven patients had to be excluded for motion artefacts. Absolute immobilization of the limb is required during the procedure, but in a few patients, an optimal positioning was difficult to obtain, because of stiffness of the spine or joints.

It was previously argued that osteoporosis in AS affects mainly the axial skeleton [12,29,30]. The current study indicates, nevertheless, that axial and peripheral trabecular bone loss are connected in AS. Spinal and peripheral trabecular vBMD were strongly correlated. Decreasing lumbar vBMD measured with QCT was, in addition, associated with deteriorated peripheral bone microarchitecture, such as thinner trabeculae, higher trabecular separation, and reduced cortical thickness. This may explain the increased frequency of hip and peripheral fractures observed in an earlier study on Swedish AS patients [5].

A plausible reason for the coupling of trabecular bone loss in the axial and peripheral skeleton is that general inflammation in AS with systemic elevation of cytokines, such as TNF-α, IL-1, and IL-6, affects osteoclasts and osteoblasts in all bone tissue. Trabecular bone loss in the vertebral bodies may, in addition, be enhanced by local inflammation and alterations of loading due to syndesmophytes and ankylosis.

In 1997, a study on 14 men with AS showed that cancellous bone volume from iliac crest biopsies correlated well with lumbar spine BMD measured with QCT, but not with DXA. The trabecular thickness in the biopsies of the AS patients was found to be lower than the reference values reported in the literature [31]. In contrast, another study reported no correlation between vBMD measured in the forearm with peripheral QCT (pQCT) and QCT values of the lumbar spine, and pQCT revealed only a few cases of osteopenia and osteoporosis. However, the patients were younger than in the present study (mean, 40 versus 49 years), and both sexes were included [7]. HRpQCT and pQCT assess different aspects of bone quality, which may also explain the discrepancy between the studies; HRpQCT measures predominantly trabecular bone in greater detail and in a more distal part of the radius and tibia, whereas pQCT gives a better evaluation of cortical bone.

Trabecular number was not associated with lumbar vBMD or age in the present study. Similarly, studies on age-related changes in the general population have shown decreased trabecular thickness, but preserved trabecular number in men, in contrast to loss of trabecular number and increased trabecular separation in women [32,33].

The current study also shows that AS patients with vertebral fractures have worse bone microarchitecture and lower vBMD in both the axial and peripheral skeleton than do age-matched AS controls. In this aspect, the situation in AS seems comparable to that in postmenopausal women and men aged older than 50 years, where vertebral fractures also have been shown to be associated with poor peripheral bone microarchitecture [34-36]. We identified cortical thinning and low cross-sectional area in the peripheral skeleton as factors strongly associated with vertebral fractures. Interestingly, another study on 920 men from the general population aged older than 50 also indicated that low cortical thickness and low cortical density in both the ultradistal radius and tibia were associated with vertebral fractures [34].

We found that the presence of syndesmophytes was associated with lower trabecular vBMD but increasing cortical vBMD in the lumbar spine. The increasing cortical vBMD was presumably reflecting pathologic new-bone formation in the cortex of the vertebral bodies. Our findings are supported by an earlier study also reporting decreasing trabecular but increasing cortical vBMD in the lumbar spine measured with QCT in AS patients in advancing stages of ankylosis [37]. The pathologic new-bone formation in the spine was, however, not coupled with any signs of hyperostosis in the peripheral microarchitecture. In contrast, it was associated with lower peripheral trabecular density and thinner trabeculae. The findings support the concept of osteoproliferation as being a local anabolic bone response to inflammation, mechanical stress, or microdamage, but not a systemic process in AS.

In the present study, lateral lumbar BMD and estimated vBMD by DXA correlated well with QCT measures of both cortical and trabecular BMD. QCT was more sensitive than AP DXA in revealing reduced BMD in the lumbar spine. We recently published data showing that lateral DXA scanning with estimation of vBMD in comparison with AP DXA is more sensitive in detecting reduced BMD, less affected by the pathologic new-bone formation in the spine, and better associated with vertebral fractures [13]. QCT has hitherto been considered to be the preferred method for measuring lumbar BMD in AS, but radiation doses and availability are, however, problems of QCT. We propose that lateral lumbar DXA with estimation of vBMD may be a valuable alternative for the assessment of lumbar BMD in AS.

The lack of a Swedish control group for the HRpQCT measurements is a limitation of the study. The healthy controls were, however, matched for age, height, weight, and BMI, and Olmsted county in Minnesota has a high proportion of inhabitants of Scandinavian descent. Other limitations are the relatively small reference population for lumbar QCT and the absence of a reference-population database for men regarding lateral lumbar DXA.

## Conclusions

The HRpQCT measurements revealed lower vBMD in the ultradistal radius and tibia in the AS patients compared with the healthy controls. Low-lumbar vBMD, vertebral fractures, and chronic AS-related changes in the spine were associated with lower vBMD and worse bone microarchitecture in the peripheral skeleton. The results indicate that osteoporosis in AS is a general process affecting both the central and the peripheral skeleton, whereas pathologic new-bone formation is localized.

## Abbreviations

AP: Anteroposterior; AS: Ankylosing spondylitis; ASDAS: Ankylosing spondylitis disease activity acore; BASDAI: Bath ankylosing spondylitis disease activity score; BASFI: Bath ankylosing spondylitis functional index; BAS-G: Bath ankylosing spondylitis patient global score; BASMI: Bath ankylosing spondylitis metrology index; BMD: Bone mineral density; BMI: Body mass index; BV/TV: Trabecular bone volume fraction; Cort: Cortical; CortCSA: Cortical cross-sectional area peripheral bone; CortPm: Cortical periosteal circumference peripheral bone; CortTh: Cortical thickness peripheral bone; CRP: C-reactive protein; CtPo: Cortical porosity; CtPoDiam: Mean cortical pore diameter; CV: Coefficient of variation; DCort: Volumetric BMD of cortical peripheral bone; DMARD: Disease-modifying antirheumatic drug; DTrab: Volumetric BMD of trabecular peripheral bone; DXA: Dual-energy x-ray absorptiometry; ESR: Erythrocyte sedimentation rate; HRpQCT: High-resolution peripheral quantitative computed tomography; mSASSS: Modified stoke ankylosing spondylitis spine score; NSAID: Nonsteroidal antiinflammatory drug; QCT: Quantitative computed tomography; TbN: Trabecular number peripheral bone; TbSp: Trabecular separation peripheral bone; Tbth: Trabecular thickness peripheral bone; TNF: Tumor necrosis factor; Totarea: Total bone area peripheral bone; Trab: Trabecular; vBMD: Volumetric bone mineral density; VOI: Volume of interest.

## Competing interests

The authors declare that they have no competing interests.

## Authors’ contributions

Study design was performed by EK, HF, and HC. EK, HF, ML, DM, JG, MG, and HC conducted the study. Data were collected by EK, HF, ML, DM, JG, MG, CO, SK, and EA. Data analysis was performed by EK, HF, ML EA, and SK. Data were interpreted by EK and HF. EK drafted the manuscript, and EK revisedmanuscript content. EK, HF, ML, JG, DM, MG, CO, EA, SK, and HC. EK and HF take responsibility for the integrity of the data analysis. All authors read and approved the final manuscript.

## References

[B1] Cooper C Carbone L Michet CJ Atkinson EJ O’Fallon WM Melton LJ 3rd Fracture risk in patients with ankylosing spondylitis: a population based study J Rheum 1994 15 1877 1882 7837154

[B2] Donnelly S Doyle DV Denton A Rolfe I McCloskey EV Spector TD Bone mineral density and vertebral compression fracture rates in ankylosing spondylitis Ann Rheum Dis 1994 15 117 121 10.1136/ard.53.2.117 8129456 PMC1005263

[B3] Mitra D Elvins DM Speden DJ Collins AJ The prevalence of vertebral fractures in mild ankylosing spondylitis and their relationship to bone mineral density Rheumatology (Oxford) 2000 15 85 89 10.1093/rheumatology/39.1.85 10662879

[B4] Vosse D Landewe R van der Heijde D van der Linden S van Staa TP Geusens P Ankylosing spondylitis and the risk of fracture: results from a large primary care-based nested case–control study Ann Rheum Dis 2009 15 1839 1842 10.1136/ard.2008.100503 19066179

[B5] Weiss RJ Wick MC Ackermann PW Montgomery SM Increased fracture risk in patients with rheumatic disorders and other inflammatory diseases: a case–control study with 53,108 patients with fracture J Rheum [Research Support, Non-U.S. Gov’t] 2010 15 2247 2250 10.3899/jrheum.100363 20889599

[B6] Robinson Y Sanden B Olerud C Increased occurrence of spinal fractures related to ankylosing spondylitis: a prospective 22-year cohort study in 17,764 patients from a national registry in Sweden Patient Saf Surg 2013 15 2 10.1186/1754-9493-7-2 23294597 PMC3571877

[B7] Karberg K Zochling J Sieper J Felsenberg D Braun J Bone loss is detected more frequently in patients with ankylosing spondylitis with syndesmophytes J Rheum 2005 15 1290 1298 15996067

[B8] van der Weijden MA van Denderen JC Lems WF Heymans MW Dijkmans BA van der Horst-Bruinsma IE Low bone mineral density is related to male gender and decreased functional capacity in early spondylarthropathies Clin Rheumatol 2011 15 497 503 10.1007/s10067-010-1538-8 20697764 PMC3062761

[B9] Gratacos J Collado A Pons F Osaba M Sanmarti R Roque M Larrosa M Munoz-Gomez J Significant loss of bone mass in patients with early, active ankylosing spondylitis: a followup study Arthritis Rheum 1999 15 2319 2324 10.1002/1529-0131(199911)42:11<2319::AID-ANR9>3.0.CO;2-G 10555026

[B10] Maillefert JF Aho LS El Maghraoui A Dougados M Roux C Changes in bone density in patients with ankylosing spondylitis: a two-year follow-up study Osteoporos Int 2001 15 605 609 10.1007/s001980170084 11527060

[B11] Jun JB Joo KB Her MY Kim TH Bae SC Yoo DH Kim SK Femoral bone mineral density is associated with vertebral fractures in patients with ankylosing spondylitis: a cross-sectional study J Rheum 2006 15 1637 1641 16881119

[B12] Ralston SH Urquhart GD Brzeski M Sturrock RD Prevalence of vertebral compression fractures due to osteoporosis in ankylosing spondylitis BMJ 1990 15 563 565 10.1136/bmj.300.6724.563 2108749 PMC1662343

[B13] Klingberg E Lorentzon M Mellstrom D Geijer M Gothlin J Hilme E Hedberg M Carlsten H Forsblad-d’Elia H Osteoporosis in ankylosing spondylitis: prevalence, risk factors and methods of assessment Arthritis Res Ther [Research Support, Non-U.S. Gov’t] 2012 15 R108 10.1186/ar3833 PMC3446485 22569245

[B14] El Maghraoui A Osteoporosis and ankylosing spondylitis Joint Bone Spine 2004 15 291 295 10.1016/j.jbspin.2003.06.002 15288853

[B15] Lange U Kluge A Strunk J Teichmann J Bachmann G Ankylosing spondylitis and bone mineral density: what is the ideal tool for measurement? Rheum Int 2005 15 115 120 10.1007/s00296-004-0515-4 15538574

[B16] Szulc P Munoz F Duboeuf F Marchand F Delmas PD Bone mineral density predicts osteoporotic fractures in elderly men: the MINOS study Osteoporos Int 2005 15 1184 1192 10.1007/s00198-005-1970-9 16096713

[B17] Schuit SC van der Klift M Weel AE de Laet CE Burger H Seeman E Hofman A Uitterlinden AG van Leeuwen JP Pols HA Fracture incidence and association with bone mineral density in elderly men and women: the Rotterdam Study Bone 2004 15 195 202 10.1016/j.bone.2003.10.001 14751578

[B18] Fields AJ Eswaran SK Jekir MG Keaveny TM Role of trabecular microarchitecture in whole-vertebral body biomechanical behavior J Bone Miner Res 2009 15 1523 1530 10.1359/jbmr.090317 19338454 PMC2730926

[B19] Seeman E The growth and age-related origins of bone fragility in men Calcif Tissue Int 2004 15 100 109 15383923 10.1007/s00223-004-0289-4

[B20] Fields AJ Nawathe S Eswaran SK Jekir MG Adams MF Papadopoulos P Keaveny TM Vertebral fragility and structural redundancy J Bone Miner Res 2012 15 2152 2158 10.1002/jbmr.1664 22623120 PMC3440513

[B21] van der Linden S Valkenburg HA Cats A Evaluation of diagnostic criteria for ankylosing spondylitis: a proposal for modification of the New York criteria Arthritis Rheum 1984 15 361 368 10.1002/art.1780270401 6231933

[B22] Burghardt AJ Kazakia GJ Ramachandran S Link TM Majumdar S Age- and gender-related differences in the geometric properties and biomechanical significance of intracortical porosity in the distal radius and tibia J Bone Miner Res [Research Support, N.I.H., Extramural] 2010 15 983 993 10.1359/jbmr.091104 PMC3153365 19888900

[B23] Nishiyama KK Macdonald HM Buie HR Hanley DA Boyd SK Postmenopausal women with osteopenia have higher cortical porosity and thinner cortices at the distal radius and tibia than women with normal aBMD: an in vivo HR-pQCT study J Bone Miner Res [Comparative Study Research Support, Non-U.S. Gov’t Validation Studies] 2010 15 882 890 10.1359/jbmr.091020 19839766

[B24] Laib A Hauselmann HJ Ruegsegger P In vivo high resolution 3D-QCT of the human forearm Technol Health Care 1998 15 329 337 10100936

[B25] Burghardt AJ Buie HR Laib A Majumdar S Boyd SK Reproducibility of direct quantitative measures of cortical bone microarchitecture of the distal radius and tibia by HR-pQCT Bone [Research Support, N.I.H., Extramural Research Support, Non-U.S. Gov’t] 2010 15 519 528 10.1016/j.bone.2010.05.034 PMC2926164 20561906

[B26] Bouxsein ML Boyd SK Christiansen BA Guldberg RE Jepsen KJ Muller R Guidelines for assessment of bone microstructure in rodents using micro-computed tomography J Bone Miner Res [Review] 2010 15 1468 1486 10.1002/jbmr.141 20533309

[B27] Genant HK Wu CY van Kuijk C Nevitt MC Vertebral fracture assessment using a semiquantitative technique J Bone Miner Res 1993 15 1137 1148 8237484 10.1002/jbmr.5650080915

[B28] Creemers MC Franssen MJ van’t Hof MA Gribnau FW van de Putte LB van Riel PL Assessment of outcome in ankylosing spondylitis: an extended radiographic scoring system Ann Rheum Dis 2005 15 127 129 10.1136/ard.2004.020503 15051621 PMC1755183

[B29] Sarikaya S Basaran A Tekin Y Ozdolap S Ortancil O Is osteoporosis generalized or localized to central skeleton in ankylosing spondylitis? J Clin Rheumatol 2007 15 20 24 10.1097/01.rhu.0000255688.83037.42 17278944

[B30] Toussirot E Michel F Wendling D Bone density, ultrasound measurements and body composition in early ankylosing spondylitis Rheumatology (Oxford) 2001 15 882 888 10.1093/rheumatology/40.8.882 11511757

[B31] Lee YS Schlotzhauer T Ott SM van Vollenhoven RF Hunter J Shapiro J Marcus R McGuire JL Skeletal status of men with early and late ankylosing spondylitis Am J Med 1997 15 2333 2341 10.1016/s0002-9343(97)00143-5 9316556

[B32] Amin S Khosla S Sex- and age-related differences in bone microarchitecture in men relative to women assessed by high-resolution peripheral quantitative computed tomography J Osteoporos 2012 15 129760 22496983 10.1155/2012/129760 PMC3307008

[B33] Khosla S Riggs BL Atkinson EJ Oberg AL McDaniel LJ Holets M Peterson JM Melton LJ Effects of sex and age on bone microstructure at the ultradistal radius: a population-based noninvasive in vivo assessment J Bone Miner Res [Comparative Study Research Support, N.I.H., Extramural] 2006 15 124 131 10.1359/JBMR.050916 PMC1352156 16355281

[B34] Szulc P Boutroy S Vilayphiou N Chaitou A Delmas PD Chapurlat R Cross-sectional analysis of the association between fragility fractures and bone microarchitecture in older men: the STRAMBO study J Bone Miner Res 2011 15 1358 1367 10.1002/jbmr.319 21611974

[B35] Boutroy S Bouxsein ML Munoz F Delmas PD In vivo assessment of trabecular bone microarchitecture by high-resolution peripheral quantitative computed tomography J Clin Endocrinol Metab 2005 15 6508 6515 10.1210/jc.2005-1258 16189253

[B36] Sornay-Rendu E Boutroy S Munoz F Delmas PD Alterations of cortical and trabecular architecture are associated with fractures in postmenopausal women, partially independent of decreased BMD measured by DXA: the OFELY study J Bone Miner Res 2007 15 425 433 10.1359/jbmr.061206 17181395

[B37] Lange U Teichmann J Strunk J Muller-Ladner U Schmidt KL Association of 1.25 vitamin D3 deficiency, disease activity and low bone mass in ankylosing spondylitis Osteoporos Int 2005 15 1999 2004 10.1007/s00198-005-1990-5 16172800

